# Bronchoscopic Intrapulmonary Recombinant Factor VIIa for Diffuse Alveolar Hemorrhage-induced Acute Respiratory Failure in MPO-ANCA Vasculitis: A Case Report

**DOI:** 10.2478/jccm-2022-0004

**Published:** 2022-05-12

**Authors:** Illaa Smesseim, Titia Schaepman-Ruys, Jan Willem Duitman, Yosta Vegting, Jorinde Raasveld, Marc Hilhorst, Alexander Vlaar, Josien van Es, Peter Bonta

**Affiliations:** 1 Department of Respiratory Medicine, Amsterdam University Medical Center, University of Amsterdam and Free University Amsterdam, Amsterdam The Netherlands; 2 Department of Experimental Immunology (EXIM), Amsterdam University Medical Center, University of Amsterdam, Amsterdam The Netherlands; 3 Department of Internal Medicine, Section of Nephrology, Location Meibergdreef Amsterdam UMC, University of Amsterdam, Amsterdam The Netherlands; 4 Department of Intensive Care Medicine, Amsterdam University Medical Centers, University of Amsterdam, Amsterdam The Netherlands

**Keywords:** ARDS, diffuse alveolar hemorrhage, DAH, novoseven, vasculitis

## Abstract

**Introduction:**

Diffuse alveolar haemorrhage (DAH) is a potentially life-threatening disease, characterized by diffuse accumulation of red blood cells within the alveoli. It can be caused by a variety of disorders. In case DAH results in severe respiratory failure, veno-venous extracorporeal membrane oxygenation (VV-ECMO) can be required. Since VV-ECMO coincides with the need for anticoagulation therapy, this results in a major clinical challenge in DAH patients with hemoptysis.

**Case presentation:**

We report a patient case with severe DAH-induced acute respiratory failure and hemoptysis in need for VV-ECMO complicated by life-threatening membrane oxygenator thrombosis. The DAH-induced hemoptysis was successfully treated with local bronchoscopic recombinant factor VIIa (rFVIIa), allowing systemic anticoagulation to prevent further membrane oxygenator thrombosis. Neither systemic clinical side effects nor differences in the serum coagulation markers occurred after applying recombinant factor VIIa (rFVIIa) treatment endobronchially.

**Conclusion:**

This is, to our knowledge, the first case that reports the use of rFVIIa in a patient with DAH due to vasculitis and in need for VV-ECMO complicated by membrane oxygenator thrombosis.

## Introduction

Diffuse alveolar haemorrhage (DAH) is a potentially life-threatening disease, characterized by diffuse accumulation of red blood cells within the alveoli [1]. As such, it can present with hemoptysis, fever and hypoxic respiratory failure and has several causes including auto-immune diseases, drug use and infection [2,3]. Treatment of DAH consists of treating the underlying cause and includes corticosteroids often combined with other immunosuppressive agents [4]. In case DAH causes severe respiratory failure, veno-venous extracorporeal membrane oxygenation (VV-ECMO) can be required. However, VV-ECMO coincides with the need for anticoagulation therapy. This is a major clinical challenge, especially in case of clinical relevant hemoptysis [5].

## Case presentation

A 47-year old female, non-smoker, with a history of a mixed connective tissue disease, presented to the emergency department of a general hospital with dyspnea and hemoptysis. She had a tachycardia of 119 beats per minute, respiratory rate of 45 per minute, and pulse oximetry of 96% with a non-rebreathing-mask (O_2_ 15L/min). Physical exam revealed red conjunctivae, signs of respiratory distress and peripheral edema without jugular vein distension. Laboratory parameters were remarkable for: hemoglobin level of 2,1 mmol/l (with negative hemolysis markers), creatinine 562 μmol/l, creatinine kinase 7172 U/L, C-reactive protein of 152 mg/L. Because of deterioration in oxygenation the patient was intubated at the emergency department and admitted to the intensive care unit (ICU). Chest computed tomography (CT) showed bilateral consolidations and ground-glass opacities. During subsequent bronchoscopy with broncho-alveolar lavage (BAL) to exclude pulmonary infections, diffuse hemorrhagic fluid was reported. Because of acute oliguric renal failure and rhabdomyolysis the patient was treated with aggressive fluid resuscitation to restore renal perfusion. Moreover, awaiting the BAL cultures, the patient received antibiotic therapy to treat a pulmonary infection and blood transfusion to treat anemia. On day 4 autoimmune serology showed a p-ANCA pattern with myeloperoxidase (MPO) antibodies; the BAL was negative for pulmonary infections. The diagnosis of MPO-ANCA associated vasculitis was established with pulmonary, renal and ocular involvement.

Treatment was escalated to high dose methylprednisolone 1000mg for 3 days and continued with plasma exchange three days after ICU admission. Nevertheless, respiratory failure and hemoptysis worsened and she was transferred to a tertiary ICU for VV-ECMO. A new CT-scan showed progressive bilateral consolidations with diffuse ground glass opacities. Hereafter, plasmapheresis, cyclophosphamide and VV-ECMO were started. A second bronchoscopy with sequential BAL was performed, and confirmed progressive hemorrhagic fluid in the sequential BAL aliquots, compatible with DAH. Standard antithrombotic therapy for VV-ECMO was postponed because of progressive hemoptysis, resulting in the need for frequent endotracheal clearance and severe underlying anemia. Unfortunately, after three days of VV-ECMO, clot formation occurred in the oxygenator’s membrane.

The combination of DAH with hemoptysis and membrane clotting oxygenator thrombosis posed a major clinical challenge in a patient dependent on VV-ECMO. Therefore, it was decided to escalate the treatment with bronchoscopic application of recombinant factor VIIa (Novoseven) and subsequently start systemic anticoagulation (unfractioned heparin) to treat and prevent further oxygenator membrane clotting; 50 μg/kg activated recombinant factor VIIa (rFVIIa) in 50 ml of 0.9% of sodium chloride was administered through the working channel of the bronchoscope [6]. A total of 12.5 ml was administered in each of the main lobar bronchi.

**Fig. 1:**
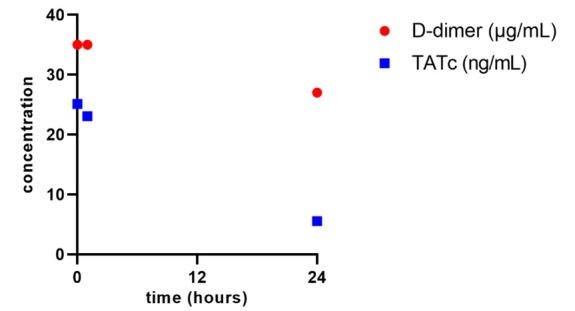
Plasma levels of D-dimer (red circles) and thrombin-antithrombin complexes (TATc; blue squares) 0, 1 and 24 hours upon first recombinant factor VIIa administration.

In order to monitor systemic coagulation activation as a side effect of bronchoscopic rFVIIa administration, the plasma D-dimer and thrombin-antithrombin complexes (TATc) levels were determined just prior to and after 1 and 24 hours. The TATc levels were also measured in the BAL fluid. As shown in Figure 1, no increase in both serum and BAL coagulation markers was observed 1 hour after local rFVIIa administration. In contrast, levels of these coagulation markers were even decreased. Upon the first rFVIIa administration hemoptysis diminished, but was still present with a reduction in need of endotracheal clearance. Therefore, the bronchoscopic procedure with administration of rFVIIa was repeated after 24 hours, after which hemoptysis resolved.

After 72 hours a bronchoscopy was repeated and showed lower red blood cell counts. In the BAL fluid increased TATc levels were observed between the first and third bronchoscopic procedure (t = 0h: 64.7 and t = 72h: 80.0 ng/mL respectively). This and the fact that hemoptysis had stopped suggested that coagulation was activated due to the administration of rFVIIa.

Over the next twelve days further improvement of pulmonary and circulatory functions was observed, oxygenator membrane clotting diminished, and the patient was successfully weaned off VV-ECMO. Eighteen days after referral to our hospital, the patient was discharged from the ICU.

## Discussion

We describe a case of a patient with DAH-induced acute respiratory failure and membrane clotting oxygenator thrombosis in need for VV-ECMO. Local bronchoscopic treatment with rFVIIa was started to treat life-threatening persistent hemoptysis. This is the first case report that reports stable – decreasing D-dimer and TATc plasma levels upon rFVIIa administration in a patient with DAH due to vasculitis and in need for VV-ECMO.

rFVIIa was originally developed for treating hemophilic patients. Furthermore, it has been successfully used to treat patients with hemorrhage during major surgery, trauma and hepatic failure [7]. The rFVIIa-tissue factor complex causes fibrin formation [8]. Because tissue factor is expressed in the alveoli during inflammation, such as pulmonary vasculitis, we believe that intrapulmonary administration of rFVIIa can contribute to local coagulation activation and subsequent resolution of the hemoptysis due to DAH as illustrated by this case report and substantiated by plasma and BAL coagulation markers over time [1]. Although the costs of rFVIIa is significant, they are nowhere as high as the costs of a prolonged ICU stay with VV-ECMO support.

## Conclusion

Bronchoscopic intrapulmonary administration of rFVIIa can be considered as a treatment option in patients with life-threatening respiratory failure due to DAH and persistent hemoptysis, with unsatisfactory response to immunosuppressive treatment of the underlying disease.
